# Gaze Following in Ungulates: Domesticated and Non-domesticated Species Follow the Gaze of Both Humans and Conspecifics in an Experimental Context

**DOI:** 10.3389/fpsyg.2020.604904

**Published:** 2020-11-19

**Authors:** Alina Schaffer, Alvaro L. Caicoya, Montserrat Colell, Ruben Holland, Conrad Ensenyat, Federica Amici

**Affiliations:** ^1^Behavioral Ecology Research Group, Institute of Biology, University of Leipzig, Leipzig, Germany; ^2^Department of Clinical Psychology and Psychobiology, Faculty of Psychology, University of Barcelona, Barcelona, Spain; ^3^Institute of Neurosciences, University of Barcelona, Barcelona, Spain; ^4^Zoo Leipzig, Leipzig, Germany; ^5^Barcelona Zoo, Barcelona, Spain; ^6^Research Group “Primate Behavioral Ecology,” Department of Human Behavior, Ecology and Culture, Max Planck Institute for Evolutionary Anthropology, Leipzig, Germany

**Keywords:** domestication, gaze following, social cognition, ungulates, human relation to animals

## Abstract

Gaze following is the ability to use others’ gaze to obtain information about the environment (e.g., food location, predators, and social interactions). As such, it may be highly adaptive in a variety of socio-ecological contexts, and thus be widespread across animal taxa. To date, gaze following has been mostly studied in primates, and partially in birds, but little is known on the gaze following abilities of other taxa and, especially, on the evolutionary pressures that led to their emergence. In this study, we used an experimental approach to test gaze following skills in a still understudied taxon, ungulates. Across four species (i.e., domestic goats and lamas, and non-domestic guanacos and mouflons), we assessed the individual ability to spontaneously follow the gaze of both conspecifics and human experimenters in different conditions. In line with our predictions, species followed the model’s gaze both with human and conspecific models, but more likely with the latter. Except for guanacos, all species showed gaze following significantly more in the experimental conditions (than in the control ones). Despite the relative low number of study subjects, our study provides the first experimental evidence of gaze following skills in non-domesticated ungulates, and contributes to understanding how gaze following skills are distributed in another taxon—an essential endeavor to identify the evolutionary pressures leading to the emergence of gaze following skills across taxa.

## Introduction

Gaze following is the ability of looking where others are looking (Butterworth and Jarrett, 1991; Emery et al., 1997). This ability is often considered one of the most basic forms of social cognition, as it allows individuals to socially acquire relevant information about the environment (e.g., about food location, presence of predators, occurrence of social interactions among group members) (Tomasello et al., 1998, 2001) and also about others’ interests and goals (Baron-Cohen, 1995). Therefore, gaze following might be highly adaptive for humans and other animals (Brooks and Meltzoff, 2002).

To date, gaze following has indeed been reported in a variety of taxa, including dogs (*Canis familiaris*) (Miklösi et al., 1998; Range and Virányi, 2011; Téglás et al., 2012; Met et al., 2014; Duranton et al., 2017), birds (Watve et al., 2002; Bugnyar et al., 2004; Schloegl et al., 2007; Goossens et al., 2008; Jaime et al., 2009; Loretto et al., 2010; Kehmeier et al., 2011; Schmidt et al., 2011; Tornick et al., 2011; also see Kaplan, 2011; Nawroth et al., 2017), reptiles (Wilkinson et al., 2010; Simpson and O’Hara, 2019), and several primate species (e.g., Itakura, 1996; Emery et al., 1997; Tomasello et al., 1998; Anderson and Mitchell, 1999; Scerif et al., 2004; Bräuer et al., 2005; Burkart and Heschl, 2006; Shepherd and Platt, 2008; Ruiz et al., 2009; see Rosati and Hare, 2009, for a review; Sandel et al., 2011; Liebal and Kaminski, 2012; Chen et al., 2017; Drayton and Santos, 2017).

Clearly, gaze following does not necessarily imply complex cognition. Povinelli and Eddy, 1996, for instance, distinguished a low-level from a high-level form of gaze following in animals (also referred to as gaze following into space versus geometrical gaze following; see Loretto et al., 2010). In particular, low-level gaze following would be an innate response triggered by a shift in the individual’s attention toward an external target: when a conspecific turns the head, for instance, the individual attention would be caught by this movement, and the individual would simply look in that direction, without any cognitive skills being involved. In contrast, high-level gaze following would also imply the ability to take others’ perspective and thus understand what others see from their location: if the individual sees a conspecific looking in another direction, for example, it might use the conspecific’s gaze as a cue to obtain information about the environment, eventually moving around barriers to gain the conspecific’s perspective (Povinelli and Eddy, 1996).

Although gaze following appears to be widespread across taxa, at least in its lower-level form, some studies have shown important differences in gaze following behavior even among closely related species (Kano and Call, 2014). In particular, species can differ from each other in two main ways. First, they can differ in their general sensitivity to gaze following: while some species reliably follow others’ gaze, others might be less sensitive to the gaze of others, and less reliably follow it. Stump-tailed macaques (*Macaca arctoides*), for instance, follow the gaze of conspecifics more frequently than other macaque species (Tomasello et al., 1998), while bonobos (*Pan paniscus*) are more likely to follow others’ gaze, compared to chimpanzees (*Pan troglodytes*) (Herrmann et al., 2010; Kano and Call, 2014). Similarly, some species might avoid direct gaze and gaze following (see Kaplan and Rogers, 2002). Second, species can specifically differ in their ability to follow the gaze of individuals of other species (i.e., allospecifics). While the gaze of a conspecific might provide relevant information to individuals in most species (so that they would benefit from following it), allospecifics’ gaze might less likely trigger gaze following behavior (see Kano and Call, 2014).

The reasons for these interspecific differences, however, are yet unclear. Some researchers, for instance, have proposed that differences in gaze following skills might depend on differences in motivation and/or selective interest in certain models (Kano and Call, 2014). Other researchers have rather highlighted the role of domestication in the emergence of gaze following skills (see Hemmer, 1990; Kaminski et al., 2005). On the one hand, domestication might reduce sensitivity to predators (because humans protect domesticated animals against other predators; Hemmer, 1990), so that gaze following might be less frequent in domesticated species, if its main function is the acquisition of information about the presence of predators (see Kaminski et al., 2005). On the other hand, domestication might have selected for especially tame and socially skilled individuals (e.g., Hare et al., 2002; Hare and Tomasello, 2005), which might have enhanced social cognitive skills, and also be better at following others’ gaze. However, while some studies have suggested that domestication has a positive effect on species’ ability to follow others’ gaze (e.g., Kaminski et al., 2004), other researchers have found no positive effect of domestication on gaze following skills (e.g., Werhahn et al., 2016). Therefore, the effect of domestication on gaze following is yet unclear, and more comparative studies are required to better understand which factors best predict interspecific variation in gaze following (Kano and Call, 2014).

In this study, we aimed to compare species in their ability to follow the gaze of conspecifics and allospecifics and, in particular, the effect of domestication on these skills. For this purpose, we tested four different ungulate species: two domesticated ones (i.e., goats, *Capra aegagrus hircus*, and lamas, *Lama glama*), and two non-domesticated ones (i.e., mouflons, *Ovis orientalis orientalis*, and guanacos, *Lama guanicoe*). We selected ungulates for two main reasons. First, ungulates are a still largely understudied taxon, with only one species yet having been tested for its gaze following skills (Kaminski et al., 2005), to our knowledge. Therefore, testing these species can significantly increase the range of species on which we have information and help to shed light on the selective pressures that might affect the emergence of gaze following skills in different taxa. Second, ungulates include a variety of domesticated and non-domesticated species, with an impressive variety of socio-ecological characteristics (see Shultz and Dunbar, 2006). Therefore, they constitute an ideal model to contrast different evolutionary hypotheses on the emergence of gaze following skills.

Here, we used a consolidated experimental approach in which subjects observed either a conspecific or a human experimenter suddenly turning the head toward a distant location. We monitored whether subjects followed the conspecific’s and the human’s gaze, by turning the head in the same direction of the model, and whether species differed in their performance. We predicted that (1) all species would more likely follow the gaze of a conspecific (rather than a human), as individuals in all species should have more interest/motivation to obtain information from conspecifics than allospecifics (see Kano and Call, 2014). Moreover, we predicted that (2) both domesticated and non-domesticated species would show gaze following skills, as also shown in other taxa (e.g., Loretto et al., 2010; Wilkinson et al., 2010; Werhahn et al., 2016).

## Materials and Methods

### Ethics

The Barcelona and Leipzig Zoos controlled and approved all the procedures. We used no invasive methods; individuals were never separated from their group and participated on a completely voluntary basis. During the task, individuals were never food or water deprived, and the tasks did not present any risks or adverse effects. Therefore, no formal approval was required.

### Subjects

We tested 17 goats (*C. aegagrus hircus*) and 3 lamas (*L. glama*) housed at the Leipzig Zoo, and 4 guanacos (*L. guanicoe*), and 4 mouflons (*Ovis aries musimon*) housed at the Barcelona Zoo. Lamas and guanacos are phylogenetically closely related, and so are goats and mouflons, with lamas and goats having been domesticated approximately 5,000–3,800 and more than 10,000 years ago, respectively (see Vigne et al., 2005; Goñalons, 2008).

Study subjects included both males and females, and were all adults (i.e., older than one year), except for the goat sample, which also included four infants (for more details on the study subjects, see Table 1). The study subjects had little experience with experimental procedures: the lamas and some of the goats had been previously tested in a neophobia test (i.e., in which individuals were provided with food close to a novel object), while the guanacos and mouflons had never been taken part in any experiment. The tasks were carried out in the external facilities of the species, and their usual management was not changed due to our tasks. While goats and lamas are commonly considered domesticated species (Zeder and Hesse, 2000; Dong et al., 2015; Diaz-Lameiro, 2016), mouflons and guanacos are not (Lincoln, 1990; Cartajena et al., 2007; Chessa et al., 2009; Yacobaccio and Vilá, 2016).

**TABLE 1:** For each species, subjects participating in the task, including their sex and age class, and the number of trials in which they participated, for each task (conspecific and human) and condition (experimental and control).

Species	Subject	Age class	Sex	Number of administered trials			
				Consp. (exper.)	Consp. (control)	Human (exper.)	Human (control)
Goat	1	Adult	Female	3	3	4	6
	2	Adult	Female	0	0	3	2
	3	Adult	Female	0	0	5	5
	4	Adult	Female	0	0	4	4
	5	Infant	Female	0	0	6	6
	6	Infant	Female	0	0	5	2
	7	Adult	Female	1	1	5	6
	8	Adult	Female	0	0	6	6
	9	Adult	Female	1	1	6	6
	10	Adult	Female	6	3	6	5
	11	Infant	Male	0	0	2	2
	12	Adult	Female	0	0	6	6
	13	Adult	Female	4	4	5	5
	14	Infant	Male	0	0	4	5
	15	Adult	Female	1	3	3	5
	16	Adult	Female	1	2	6	6
	17	Adult	Male	1	1	6	6
Guanaco	Hembra abajo	Adult	Female	7	6	6	8
	Hembra arriba	Adult	Female	5	13	10	10
	Rojo	Adult	Male	6	7	8	8
	Verde	Adult	Male	6	6	7	8
Lama	Flax	Adult	Male	3	2	6	6
	Krümel	Adult	Male	3	3	6	6
	Sancho	Adult	Male	1	2	6	6
Mouflon	Circulo amarillo	Adult	Female	6	7	8	6
	Circulo naranja	Adult	Female	1	2	9	9
	Cuadrado blanco	Adult	Female	9	11	8	11
	Cuadrado rojo	Adult	Female	8	6	7	7
	Cuadrado verde	Adult	Female	6	4	9	12
	Macho	Adult	Male	6	4	7	10

### Procedures

We administered two tasks, one using as a model a conspecific (Conspecific task), and one a human experimenter (Human task). We originally aimed to administer 6 to 12 trials per task and condition (i.e., Experimental and Control), but as subjects differed in their motivation to participate, the number of trials administered in each task and condition varied across them (see Table 1). Subjects were tested when they were approximately 1 to 4 m from the experimenter. All trials were video recorded with a video camera positioned just outside the ungulate enclosure, so that the subject was clearly visible. Subject responses were later coded from the videos (see below).

**FIGURE 1:**
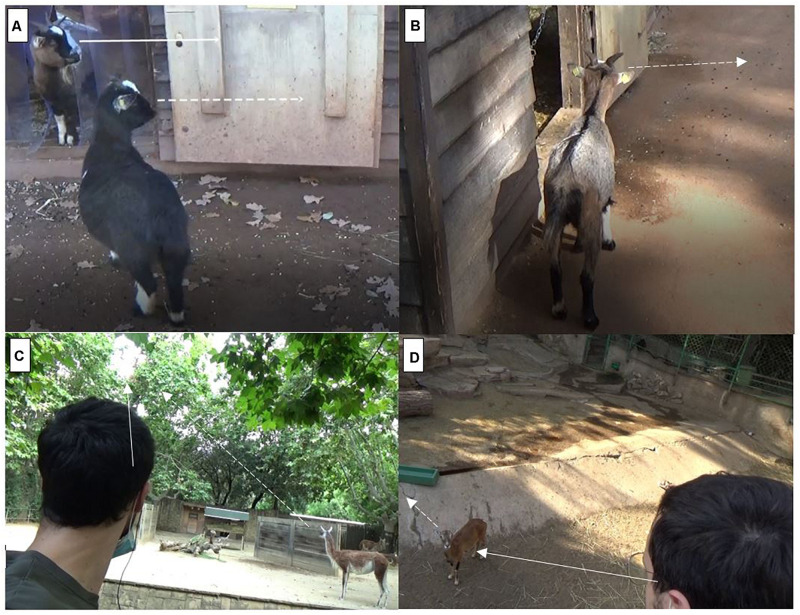
Experimental setup for the two tasks and conditions: **(A)** Conspecific experimental trial. **(B)** Conspecific control trial. **(C)** Human experimental trial. **(D)** Human control trial. Continuous lines indicate the model’s gaze direction, while dotted lines indicate subjects’ gaze direction when trials were coded as positive.

In the experimental condition of the conspecific task, we opportunistically waited for two individuals facing each other, one giving its back to the experimenter (i.e., subject) and one having the experimenter in his visual field (i.e., model; see Figure 1A). The experimenter tried to catch the model’s attention (e.g., holding a piece of food in the air), so that the model would visibly move his head in another direction (e.g., raising or turning his head toward the experimenter), while the subject looked toward the model (i.e., so that the subject could see the model move his head). When the model moved the head toward the experimenter and the subject looked at the model, a trial was started. The control condition of the conspecific task was identical, except that no model was present, and the trial was started when the subject was giving his back to the experimenter (so that the subject was provided a no gaze cue; see Figure 1B). Trials were scored as successful if the subject turned his head in the same direction (i.e., at least 45°) in which the model looked at (for the control trials, in the direction in which the model looked at in the corresponding experimental trial).

In the experimental condition of the human task, we opportunistically waited for an individual (i.e., subject) to look at the experimenter (i.e., model; see Figure 1C). The model then suddenly raised his/her head toward a distant upper corner of the enclosure (either on the right or on the left, randomizing the side across subjects and trials), and a trial was started. The control condition of the human task was identical, except that the model raised his/her head toward the body of the subject (see Figure 1D). Trials were scored as successful if the subject turned his head toward the same upper corner of the enclosure (i.e., at least 45°) in which the model looked at (for the control trials, in the direction in which the model looked at in the corresponding experimental trial).

In both the conspecific and the human tasks, we first tested goats and lamas with 10-s trials. However, the greatest majority of subjects turned their head in the first 3 s of the experimental trials (i.e., 75% in lamas, 79% in goats). When testing guanacos and mouflons, therefore, we preferred to administer shorter trials (i.e., 3-s trials) to be more conservative (i.e., to avoid coding trials as positive when subjects moved the head for other reasons). Clearly, in order to ensure comparability across species, trials were coded as successful in all species and conditions if the response (see above) was given in the first 3 s. As all trials were video recorded and later scored from the videos (see above), the 3-s interval could be accurately measured from the videos.

### Statistical Analyses

Analyses were conducted using generalized linear mixed models (Baayen et al., 2008) with the glmmTMB package (version 1.0.1; Brooks et al., 2017) in R (R Core Team, version 3.5.0). Our models were run with a binomial structure, entering one line per subject and trial, and further specifying whether the trial was successful (see above), the task and condition administered, the trial number, and the species, sex, and age of the subject. A second observer independently coded 20% of the videos (i.e., whether the trial was successful), and inter-observer reliability was excellent (Cohen’s kappa = 0.94).

We then assessed whether the three-way interaction of species (as categorical predictor with four levels), task (two levels: conspecific and human) and condition (two levels: experimental and control) predicted subject’s response (i.e., whether they would direct their gaze in the direction of the model’s gaze, as explained above). In the model, we further included all the two-way interactions between species, task, and condition, and their main effects. We also included subject age and sex as controls (as in some species, gaze following skills are known to completely develop only by the end of infancy; e.g., Teufel et al., 2010; Rosati et al., 2016; and to be higher in females; e.g., Rosati et al., 2016). We finally included trial number as control (as response to others’ gaze may vary through time, either increasing as a result of learning or decreasing as a result of habituation: Schloegl et al., 2007; Loretto et al., 2010), and subject identity as random factor.

We used likelihood ratio tests (Dobson et al., 2001) to compare the full model containing all predictors with the null model containing only control predictors and random factors. When the full model significantly differed from the null model, likelihood ratio tests were conducted to obtain the *p*-values for each test predictor via single-term deletion, using the R function drop1 (Barr et al., 2013). If the three-way interaction was not significant, we removed it from the full model and re-run the comparison with the null model by only including the two-way interaction of condition with species and condition with task, their main effects, control predictors, and the random factor. We detected no convergence issues. To rule out colinearity, we determined the VIFs (Field, 2005), which were minimal (maximum VIFs = 2.01).

## Results

**FIGURE 2:**
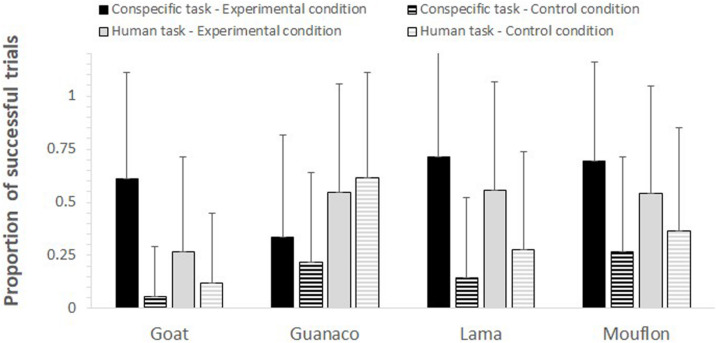
For each species, task, and condition, the mean proportion (+SD) of trials in which subjects followed the model’s gaze.

The full-null model comparison was significant (GLMM: χ^2^ = 76.61, df = 15, *p* < 0.001). The two-way interactions between condition and task (*p* < 0.001) and condition and species (*p* = 0.005) were both significant. In particular, the study subjects looked in the model’s direction more in the experimental than in the control condition in both tasks, although this difference was stronger in the conspecific task (conspecific task: *p* < 0.001; human task: *p* = 0.016; see Table 2). Moreover, while all species overall followed the model’s gaze more in the experimental than in the control condition (see Figure 2), goats (*p* < 0.001), lamas (*p* = 0.002), and mouflons (*p* < 0.001) did it significantly so, but not guanacos (*p* = 0.638).

**TABLE 2:** Summary of the results for the full model, including the reference category for categorical predictors, estimates, standard errors (SE), *z*-values (*z*), confidence intervals (CIs), and *p*-values for each test predictor (in bold, when significant) and control predictor (in italics).

Predictors	Reference category	Estimate	SE	*z*	2.5% CI	97.5% CI	*P*
Intercept		–2.95	0.48	–6.13	–3.89	–2.01	
Species	Guanaco	2.17	0.52	4.15	1.15	3.20	
	Lama	1.05	0.72	1.46	–0.36	2.45	
	Mouflon	1.63	0.47	3.48	0.71	2.54	
Condition	Experimental	2.77	0.55	5.01	1.69	3.85	
Task	Human	1.08	0.34	3.15	0.41	1.75	
Species*condition	Guanaco, experimental	–1.82	0.56	–3.23	–2.93	–0.71	0.005*
	Lama, experimental	0.00	0.75	0.00	–1.47	1.47	
	Mouflon, experimental	–0.60	0.52	–1.16	–1.63	0.42	
Task*condition	Human, experimental	–1.54	0.45	–3.44	–2.42	–0.66	< 0.001*
*Age class*	Infant	–0.86	0.64	–1.35	–2.11	0.39	0.160
*Sex*	Male	–0.07	0.36	–0.21	–0.77	0.63	0.834
*Trial*		–0.02	0.04	–0.40	–0.10	0.07	0.693

*The model had a binomial distribution and included subject identity as random effect. The asterisks denote significant *p*-values for the test predictors.*

## Discussion

Our study provides the first experimental evidence of gaze following skills in non-domesticated ungulates. In line with our predictions, ungulates followed the model’s gaze both with human and conspecific models, but were more likely to do so when the model belonged to the same species. Moreover, while all species followed the model’s gaze more in the experimental than in the control conditions, non-domesticated guanacos failed to significantly do so (but see below for a better discussion on the relatively low sample size).

The main finding of our research is that gaze following skills are present in ungulates, even in non-domesticated species (i.e., mouflons). This is in line with previous studies in other taxa, which have already shown that non-domesticated species can reliably follow others’ gaze (e.g., Loretto et al., 2010; Wilkinson et al., 2010), sometimes even better than their domesticated counterparts (e.g., Werhahn et al., 2016). Therefore, our study provides no support to the hypothesis that domesticated species show different gaze following skills than non-domesticated ones. Indeed, domesticated species do not seem to have a general advantage over non-domesticated species when following others’ gaze (as expected if close co-evolution with humans during domestication had selected for socially skilled individuals; see Hare et al., 2002). Similarly, non-domesticated species do not seem to outperform domesticated ones (as expected if gaze following skills were less adaptive in domesticated species, which receive extensive protection from predators by humans; see Kaminski et al., 2005). In contrast, gaze following appears to be really widespread across taxa, at least in its simple forms.

In contrast to non-domesticated mouflons, however, non-domesticated guanacos failed to reliably follow the model’s gaze, showing the same probability of gaze following in both experimental and control conditions. As visible in Figure 2, these results are mainly due to the low performance of guanacos (i.e., a higher proportion of successful trials in the control rather than experimental condition) when being tested with the human model. At the moment, it is not possible to understand why guanacos performed worse than the other species (including mouflons), especially with allospecific models. One reason might be that guanacos, for some yet unknown reason, show more selective attention toward their conspecifics, as chimpanzees also do (see Kano and Call, 2014). However, it is also simply possible that these results depend on our small sample size, as we could only test four guanacos. Although a larger sample size might have therefore provided different results, it is important to note that other species in our study showed evidence of gaze following skills, despite also having a small sample size (e.g., lamas, *N* = 3). Moreover, while the inclusion of more study subjects might show that also guanacos can follow the gaze of humans and conspecifics, this study already provides evidence that domestication is not necessary prerequisite for the emergence of gaze following skills in ungulates.

While it is true that guanacos performed especially poorly when tested with a human model, all species performed significantly worse when tested with humans rather than conspecifics. This seems to confirm that animals, either domesticated or not, generally have more interest and/or motivation to follow the gaze of conspecifics, as these can more likely provide relevant information (see Kano and Call, 2014). These findings have important implications for the study of interactions between humans and other animals. On the one side, they suggest an astonishing ability of most animal species (also non-domesticated ones) to use human gaze in the same way as conspecific gaze. On the other side, they suggest some limits in this ability, even in domesticated species.

Incidentally, sex, age, and trial number had no effect on individual performance in our study. These results are also largely in line with previous studies, which suggest that gaze following skills, at least in its lower-level form, emerge early on through development (see e.g., Kaminski et al., 2005; Range and Virányi, 2011). Moreover, as in previous studies (e.g., Kaminski et al., 2005), performance did not increase through time, suggesting that individual response was not the result of a learning process during the study.

Clearly, this study must be considered as a first attempt to study gaze following skills in ungulates. From a cognitive point of view, for instance, further research is needed to understand the psychological underpinnings of gaze following skills in the different species. By administering further conditions in which individuals need to take others’ perspective to follow their gaze, we might be able to better understand whether ungulate species show high- or low-level forms of gaze following (see e.g., Amici et al., 2009; Loretto et al., 2010). Furthermore, future studies should include more individuals and species, to have more power, to better control for inter-individual differences and also to test other evolutionary hypotheses on the emergence of gaze following skills (e.g., high-level forms of gaze following are more likely to emerge in species with complex sociality; see e.g., Aureli et al., 2008; Dunbar, 2009). In the future, it will be especially important to also test other non-domesticated species. The ancestors of both guanacos and mouflons, for instance, have also been domesticated (i.e., into lamas and sheep; see e.g., Goñalons, 2008; Chessa et al., 2009; Alberto et al., 2018). Therefore, it is still possible that gaze following skills in these species are linked to the favorable pre-adaptive characteristics possessed by their ancestors, which might have favored their domestication, but also the emergence of social cognitive skills like gaze following (see e.g., Zeder, 2012). Finally, future studies should assess whether ungulate species differ in their sensitivity to the gaze of humans and conspecifics, depending on the context (e.g., competitive or cooperative; see Castellano-Navarro et al., unpublished). Overall, our study confirms ungulates as a promising taxon to study comparative cognition, and zoo-housed animals as ideal subjects to extend the range of tested species, also including those that have long been neglected in cognitive research (Nawroth et al., 2017).

## Data Availability Statement

The raw data supporting the conclusions of this article will be made available by the authors, without undue reservation.

## Ethics Statement

Ethical review and approval was not required for the animal study because The Barcelona and Leipzig Zoos controlled and approved all the procedures. We used no invasive methods, individuals were never separated from their group and participated on a completely voluntary basis. During the task, individuals were never food or water deprived, and the tasks did not present any risks or adverse effects. Therefore, no formal approval was required. Written informed consent was obtained from the individual(s) for the publication of any potentially identifiable images or data included in this article.

## Author Contributions

AS and AC collected the data, with support from MC, RH, and CE. AS, AC, and FA analyzed the data and wrote the manuscript, with extensive feedback from the other authors. All the authors designed the study together and contributed to the article and approved the submitted version.

## Conflict of Interest

RH was employed by company Zoo Leipzig. The remaining authors declare that the research was conducted in the absence of any commercial or financial relationships that could be construed as a potential conflict of interest.

## References

[B1] Alberto F. J. Boyer F. Orozco-terWengel P. Streeter I. Servin B. de Villemereuil P. (2018). Convergent genomic signatures of domestication in sheep and goats. *Nat. Commun.* 9:813. 10.1038/s41467-018-03206-y PMC5840369 29511174

[B2] Amici F. Aureli F. Visalberghi E. Call J. (2009). Spider monkeys (*Ateles geoffroyi*) and capuchin monkeys (*Cebus apella*) follow gaze around barriers: evidence for perspective taking? *J. Comp. Psychol.* 123 368–374. 10.1037/a0017079 19929105

[B3] Anderson J. R. Mitchell R. W. (1999). Macaques but not lemurs co-orient visually with humans. *Folia Primatol.* 70 17–22. 10.1159/000021670 10050063

[B4] Aureli F. Schaffner C. M. Boesch C. Bearder S. K. Call J. Chapman C. A. (2008). Fission-fusion dynamics: new research frameworks. *Curr. Anthropol.* 49 627–654. 10.2307/20142694

[B5] Baayen R. H. Davidson D. J. Bates D. M. (2008). Mixed-effects modeling with crossed random effects for subjects and items. *J. Mem. Lang.* 59 390–412. 10.1016/j.jml.2007.12.005

[B6] Baron-Cohen S. (1995). *Mindblindness: An Essay on Autism and Theory of Mind (Learning, Development, and Conceptual Change).* Cambridge, MA: MIT Press.

[B7] Barr D. J. Levy R. Scheepers C. Tily H. J. (2013). Random effects structure for confirmatory hypothesis testing: keep it maximal. *J. Mem. Lang.* 68 255–278. 10.1016/j.jml.2012.11.001 24403724 PMC3881361

[B8] Bräuer J. Call J. Tomasello M. (2005). All great ape species follow gaze to distant locations and around barriers. *J. Comp. Psychol.* 119 145–154. 10.1037/0735-7036.119.2.145 15982158

[B9] Brooks M. E. Kristensen K. van Benthem K. J. Magnusson A. Berg C. W. Nielsen A. (2017). glmmTMB balances speed and flexibility among packages for zero-inflated generalized linear mixed modeling. *R J.* 9 378–400. 10.32614/rj-2017-066

[B10] Brooks R. Meltzoff A. N. (2002). The importance of eyes: how infants interpret adult looking behavior. *Dev. Psychol.* 38 958–966. 10.1037/0012-1649.38.6.958 12428707 PMC1351351

[B11] Bugnyar T. Stöwe M. Heinrich B. (2004). Ravens, *Corvus corax*, follow gaze direction of humans around obstacles. *Proc. R. Soc. B Biol. Sci.* 271 1331–1336. 10.1098/rspb.2004.2738 15306330 PMC1691735

[B12] Burkart J. Heschl A. (2006). Geometrical gaze following in common marmosets (*Callithrix jacchus*). *J. Comp. Psychol.* 120 120–130. 10.1037/0735-7036.120.2.120 16719590

[B13] Butterworth G. Jarrett N. (1991). What minds have in common is space: spatial mechanisms serving joint visual attention in infancy. *Br. J. Dev. Psychol.* 9 55–72. 10.1111/j.2044-835x.1991.tb00862.x

[B14] Cartajena I. Núñez L. Grosjean M. (2007). Camelid domestication on the western slope of the Puna de Atacama, northern Chile. *Anthropozoologica* 42 155–173.

[B15] Chen T. Gao J. Tan J. Tao R. Su Y. (2017). Variation in gaze-following between two Asian colobine monkeys. *J. Primatol.* 58 525–534. 10.1007/s10329-017-0612-0 28540427

[B16] Chessa B. Pereira F. Arnaud F. Amorim A. Goyache F. Mainland I. (2009). Revealing the history of sheep domestication using retrovirus integrations. *Science* 324 532–536. 10.1126/science.1170587 19390051 PMC3145132

[B17] Diaz-Lameiro A. M. (2016). *Evolutionary Origins and Domestication of South American Camelids, the Alpaca (Vicugna pacos) and the Llama (Lama glama) Explained through Molecular DNA Methods.* New York, NY: State University of New York System.

[B18] Dobson A. Zidek J. Lindsey J. (2001). *An Introduction to Generalized Linear Models.* London: Chapman and Hall.

[B19] Dong Y. Zhang X. Xie M. Arefnezhad B. Wang Z. Wang W. (2015). Reference genome of wild goat (*Capra aegagrus*) and sequencing of goat breeds provide insight into genic basis of goat domestication. *BMC Genomics* 16:431. 10.1186/s12864-015-1606-1 26044654 PMC4455334

[B20] Drayton L. A. Santos L. R. (2017). Do rhesus macaques, *Macaca mulatta*, understand what others know when gaze following? *Anim. Behav.* 134 193–199. 10.1016/j.anbehav.2017.10.016

[B21] Dunbar R. I. M. (2009). The social brain hypothesis and its implications for social evolution. *Ann. Hum. Biol.* 36 562–572. 10.1080/03014460902960289 19575315

[B22] Duranton C. Range F. Virányi Z. (2017). Do pet dogs (*Canis familiaris*) follow ostensive and non-ostensive human gaze to distant space and to objects? *R. Soc. Open Sci.* 4:170349. 10.1098/rsos.170349 28791164 PMC5541559

[B23] Emery N. J. Lorincz E. N. Perrett D. I. Oram M. W. Baker C. I. (1997). Gaze following and joint attention in rhesus monkeys (*Macaca mulatta*). *J. Comp. Psychol.* 111 286–293. 10.1037/0735-7036.111.3.286 9286096

[B24] Field A. (2005). *Discovering Statistics Using SPSS.* London: Sage Publications Ltd.

[B25] Goñalons G. L. M. (2008). Camelids in ancient Andean societies: a review of the zooarchaeological evidence. *Quat. Int.* 185 59–68. 10.1016/j.quaint.2007.05.022

[B26] Goossens B. M. A. Dekleva M. Reader S. M. Sterck E. H. M. Bolhuis J. J. (2008). Gaze following in monkeys is modulated by observed facial expressions. *Anim. Behav.* 75 1673–1681. 10.1016/j.anbehav.2007.10.020

[B27] Hare B. Brown M. Williamson C. Tomasello M. (2002). The domestication of social cognition in dogs. *Science* 298 1634–1636. 10.1126/science.1072702 12446914

[B28] Hare B. Tomasello M. (2005). Human-like social skills in dogs? *Trends Cogn. Sci.* 9 439–444. 10.1016/j.tics.2005.07.003 16061417

[B29] Hemmer H. (1990). *Domestication: the Decline of Environmental Appreciation.* Cambridge: Cambridge University Press.

[B30] Herrmann E. Hare B. Call J. Tomasello M. (2010). Differences in the cognitive skills of bonobos and chimpanzees. *PLoS One* 5:e12438. 10.1371/journal.pone.0012438 20806062 PMC2929188

[B31] Itakura S. (1996). An exploratory study of gaze-monitoring in nonhuman primates 1. *Jpn. Psychol. Res.* 38 174–180. 10.1111/j.1468-5884.1996.tb00022.x

[B32] Jaime M. Lopez J. P. Lickliter R. (2009). Bobwhite quail (*Colinus virginianus*) hatchlings track the direction of human gaze. *Anim. Cogn.* 12 559–565. 10.1007/s10071-009-0214-3 19205762 PMC2764337

[B33] Kaminski J. Call J. Fischer J. (2004). Word learning in a domestic dog: evidence for” fast mapping”. *Science* 304 1682–1683. 10.1126/science.1097859 15192233

[B34] Kaminski J. Riedel J. Call J. Tomasello M. (2005). Domestic goats, *Capra hircus*, follow gaze direction and use social cues in an object choice task. *Anim. Behav.* 69 11–18. 10.1016/j.anbehav.2004.05.008

[B35] Kano F. Call J. (2014). Cross-species variation in gaze following and conspecific preference among great apes, human infants and adults. *Anim. Behav.* 91 137–150. 10.1016/j.anbehav.2014.03.011

[B36] Kaplan G. (2011). Pointing gesture in a bird-merely instrumental or a cognitively complex behavior? *Curr. Zool.* 57 453–467. 10.1093/czoolo/57.4.453

[B37] Kaplan G. Rogers L. J. (2002). Patterns of gazing in orangutans (*Pongo pygmaeus*). *Int. J. Primatol.* 23 501–526.

[B38] Kehmeier S. Schloegl C. Scheiber I. B. R. Weiss B. M. (2011). Early development of gaze following into distant space in juvenile Greylag geese (*Anser anser*). *Anim. Cogn.* 14 477–485. 10.1007/s10071-011-0381-x 21308474

[B39] Liebal K. Kaminski J. (2012). Gibbons (*Hylobates pileatus*, *H. moloch*, *H. lar*, *Symphalangus syndactylus*) follow human gaze, but do not take the visual perspective of others. *Anim. Cogn.* 15 1211–1216. 10.1007/s10071-012-0543-5 22847522

[B40] Lincoln G. A. (1990). Correlation with changes in horns and pelage, but not reproduction, of seasonal cycles in the secretion of prolactin in rams of wild, feral and domesticated breeds of sheep. *Reproduction* 90 285–296. 10.1530/jrf.0.0900285 2121973

[B41] Loretto M.-C. Schloegl C. Bugnyar T. (2010). Northern bald ibises follow others’ gaze into distant space but not behind barriers. *Biol. Lett.* 6 14–17. 10.1098/rsbl.2009.0510 19656858 PMC2817240

[B42] Met A. Miklósi Á. Lakatos G. (2014). Gaze-following behind barriers in domestic dogs. *Anim. Cogn.* 17 1401–1405. 10.1007/s10071-014-0754-z 24816625

[B43] Miklösi Á. Polgárdi R. Topál J. Csányi V. (1998). Use of experimenter-given cues in dogs. *Anim. Cogn.* 1 113–121. 10.1007/s100710050016 24399275

[B44] Nawroth C. Trincas E. Favaro L. (2017). African penguins follow the gaze direction of conspecifics. *PeerJ* 5:e3459. 10.7717/peerj.3459 28626619 PMC5470578

[B45] Povinelli D. J. Eddy T. J. (1996). Chimpanzees: joint visual attention. *Psychol. Sci.* 7 129–135. 10.1111/j.1467-9280.1996.tb00345.x

[B46] Range F. Virányi Z. (2011). Development of gaze following abilities in wolves (*Canis lupus*). *PLoS One* 6:e16888. 10.1371/journal.pone.0016888 21373192 PMC3044139

[B47] Rosati A. G. Arre A. M. Platt M. L. Santos L. R. (2016). Rhesus monkeys show human-like changes in gaze following across the lifespan. *Proc. R. Soc. B Biol. Sci.* 283:20160376. 10.1098/rspb.2016.0376 27170712 PMC4874712

[B48] Rosati A. G. Hare B. (2009). Looking past the model species: diversity in gaze-following skills across primates. *Curr. Opin. Neurobiol.* 19 45–51. 10.1016/j.conb.2009.03.002 19394214

[B49] Ruiz A. Gómez J. C. Roeder J. J. Byrne R. W. (2009). Gaze following and gaze priming in lemurs. *Anim. Cogn.* 12 427–434. 10.1007/s10071-008-0202-z 19107531

[B50] Sandel A. A. MacLean E. L. Hare B. (2011). Evidence from four lemur species that ringtailed lemur social cognition converges with that of haplorhine primates. *Anim. Behav.* 81 925–931. 10.1016/j.anbehav.2011.01.020

[B51] Scerif G. Gomez J.-C. Byrne R. W. (2004). What do Diana monkeys know about the focus of attention of a conspecific? *Anim. Behav.* 68 1239–1247. 10.1016/j.anbehav.2004.01.011

[B52] Schloegl C. Kotrschal K. Bugnyar T. (2007). Gaze following in common ravens, *Corvus corax*: ontogeny and habituation. *Anim. Behav.* 74 769–778. 10.1016/j.anbehav.2006.08.017

[B53] Schmidt J. Scheid C. Kotrschal K. Bugnyar T. Schloegl C. (2011). Gaze direction–A cue for hidden food in rooks (*Corvus frugilegus*)? *Behav. Process.* 88 88–93. 10.1016/j.beproc.2011.08.002 21855614 PMC3185283

[B54] Shepherd S. V. Platt M. L. (2008). Spontaneous social orienting and gaze following in ringtailed lemurs (*Lemur catta*). *Anim. Cogn.* 11 13–20. 10.1007/s10071-007-0083-6 17492318

[B55] Shultz S. Dunbar R. I. M. (2006). Both social and ecological factors predict ungulate brain size. *Proc. R. Soc. B Biol. Sci.* 273 207–215. 10.1098/rspb.2005.3283 16555789 PMC1560022

[B56] Simpson J. O’Hara S. J. (2019). Gaze following in an asocial reptile (*Eublepharis macularius*). *Anim. Cogn.* 22 145–152. 10.1007/s10071-018-1230-y 30580392 PMC6373252

[B57] Téglás E. Gergely A. Kupán K. Miklósi Á. Topál J. (2012). Dogs’ gaze following is tuned to human communicative signals. *Curr. Biol.* 22 209–212. 10.1016/j.cub.2011.12.018 22226744

[B58] Teufel C. Gutmann A. Pirow R. Fischer J. (2010). Facial expressions modulate the ontogenetic trajectory of gaze-following among monkeys. *Dev. Sci.* 13 913–922. 10.1111/j.1467-7687.2010.00956.x 20977562

[B59] Tomasello M. Call J. Hare B. (1998). Five primate species follow the visual gaze of conspecifics. *Anim. Behav.* 55 1063–1069. 10.1006/anbe.1997.0636 9632490

[B60] Tomasello M. Hare B. Fogleman T. (2001). The ontogeny of gaze following in chimpanzees, *Pan troglodytes*, and rhesus macaques, *Macaca mulatta*. *Anim. Behav.* 61 335–343.

[B61] Tornick J. K. Gibson B. M. Kispert D. Wilkinson M. (2011). Clark’s nutcrackers (*Nucifraga columbiana*) use gestures to identify the location of hidden food. *Anim. Cogn.* 14 117–125. 20838837 10.1007/s10071-010-0349-2

[B62] Vigne J.-D. Peters J. Helmer D. (2005). *The First Steps of Animal Domestication. New Archaeozoological Approaches.* Oxford: Oxbow Books.

[B63] Watve M. Thakar J. Kale A. Puntambekar S. Shaikh I. Vaze K. (2002). Bee-eaters (*Merops orientalis*) respond to what a predator can see. *Anim. Cogn.* 5 253–259. 12461603 10.1007/s10071-002-0155-6

[B64] Werhahn G. Virányi Z. Barrera G. Sommese A. Range F. (2016). Wolves (*Canis lupus*) and dogs (*Canis familiaris*) differ in following human gaze into distant space but respond similar to their packmates’ gaze. *J. Comp. Psychol.* 130 288–298. 10.1037/com0000036 27244538 PMC5321535

[B65] Wilkinson A. Mandl I. Bugnyar T. Huber L. (2010). Gaze following in the red-footed tortoise (*Geochelone carbonaria*). *Anim. Cogn.* 13 765–769. 20411292 10.1007/s10071-010-0320-2

[B66] Yacobaccio H. D. Vilá B. L. (2016). A model for llama (*Lama glama* Linnaeus, 1758) domestication in the southern Andes. *Anthropozoologica* 51 5–13.

[B67] Zeder M. A. (2012). “Pathways to animal domestication,” in *Biodiversity in Agriculture: Domestication, Evolution, and Sustainability*, eds Gepts P. Bettinger R. L. Famula T. R. (Cambridge: Cambridge University Press), 227–259.

[B68] Zeder M. A. Hesse B. (2000). The initial domestication of goats (*Capra hircus*) in the Zagros mountains 10,000 years ago. *Science* 287 2254–2257. 10731145 10.1126/science.287.5461.2254

